# Key stakeholder opinions for a national learner education handover

**DOI:** 10.1186/s12909-019-1598-7

**Published:** 2019-05-16

**Authors:** Aliya Kassam, Mariela Ruetalo, Maureen Topps, Margo Mountjoy, Mark Walton, Susan Edwards, Leslie Nickell

**Affiliations:** 1 Cumming School of Medicine, University of Calgary, Calgary, Alberta Canada; 2 University of Toronto, Toronto, Ontario Canada; 3 McMaster University, Hamilton, Ontario Canada

## Abstract

**Background:**

Sharing information about learners during training is seen as an important component supporting learner progression and relevant to patient safety. Shared information may cover topics from accommodation requirements to unprofessional behavior. The purpose of this study was to determine the views of key stakeholders on a proposed national information sharing process during the transition from undergraduate to postgraduate medical education in Canada, termed the Learner Education Handover (LEH).

**Method:**

Key stakeholder groups including medical students, resident physicians, residency program directors, medical regulatory authority representatives, undergraduate medical education deans, student affairs leaders, postgraduate medical education deans participated in focus groups conducted via teleconference. Data were transcribed and coded independently by two coders, then analyzed for themes informed by principles of constructivist grounded theory.

**Results:**

Sixty participants (33 males and 27 females) from 16 focus groups representing key stakeholder groups participated. Most recognized value in a national LEH that would facilitate a smooth learner transition from medical school to residency. Potential risks and benefits of the LEH were identified. Themes significant to the *content*, *process* and *format* of the LEH also emerged. Guiding principles of the LEH process were determined to include that it be learner-centered while supporting patient safety, resident wellness and professional behavior. The learner and representatives from their undergraduate medical education environment would each contribute to the LEH.

**Conclusions:**

The LEH must advocate for the learner with respect for learner privacy, while promoting professionalism, patient safety and learner wellness.

## Background

Current literature suggests that medical learners feel inadequately prepared for residency, particularly in terms of clinical experience, knowledge and skills [1–3]. Faculty are often able to pinpoint where improvements are needed to help ease the transition however these are heavily based on technical deficits that need improvement and no other competency domains [2, 4]. Recent studies have developed a model [5] for an educational handover or learner handover tool [6] whereby information about the learner is shared with faculty members. These studies however did not engage the learner during the handover process.

Sozener et al. identified that there may be a disconnect between how the medical school leadership write about the student to ensure a successful match and the actual sharing of information itself and “*this concern could also lead to a healthy discussion within the medical school to reconcile potential differences between the two documents”* (Dean’s letter and information sharing/handover document) [6]. The Dean’s letter is a document sent to a residency program by a medical school (undergraduate medical education) dean which describes a candidate in his/her final year of medical school. The Dean’s letter can also be referred to as the Medical Student Performance Evaluation or Medical Student Performance Record (MSPE or MSPR) letter which accompanies medical student's residency application. This letter summarizes the student's medical school performance and provides a summary evaluation of the student's potential as a resident [7, 8].

There are differing viewpoints on information sharing about learners. On the one hand, it may help to develop better reporting tool for learners and direct teaching toward specific learner needs that may help to produce better physicians. This practice supports patient safety by ensuring that graduates have the required knowledge and skills for residency [9]. On the other hand, information sharing may lead to bias towards the student, potentially resulting in unfair advantages and disadvantages [10]. To date, there is no evidence demonstrating that information sharing has been either detrimental or useful in remediating struggling students in order to prepare them for their transition into residency [9, 11]. In the United States, only 14% of medical schools have written policies about sharing information yet 57% of clerkship directors design remediation plans for struggling students; it is unknown whether these plans focus on personal or non-academic needs of the learner [9, 11].

Two recent scoping reviews have been conducted to explore learner handovers. The first examined the influence of prior performance information on ratings of current performance for learner handovers [12]. Due to the scarcity of studies in the medical education literature, the review looked at literature across other disciplines such as psychology, business, sports, and education. When considering indirect prior performance information, the majority of studies supported an assimilation effect which means that in the context of a learner handover bias could be prevalent. Specifically, a faculty member’s perception of the learner from prior performance information (e.g., formal learner handover, a learner dashboard, or learner reputation), is biased toward the previous performance information. This would cause significant concern in the medical education community and support the arguments of those opposed to learner handover. Of more concern is the suggestion that negative prior performance information may be more influential than positive prior performance information [12].

The second scoping review [13] examined existing scholarship on learner-centered [14] initiatives to facilitate the transition from medical school to residency. While a variety of learner-centered programs that focus on specific professional competencies have been implemented to ease the transition to residency, many do not engage learners as key stakeholders in program development. The existing literature highlights various gaps in approaches particularly with respect to addressing non-medical skills competencies, and focusing on individual learners needs. Novel and innovative programs, including learner handovers, may better support the transition needs of medical residents.

Given the impact of prior performance information and the lack of learner centered interventions that exist to help learners transition from medical school to residency, it is important to explore key stakeholder opinions about a learner handover.

An Association of Faculties of Medicine of Canada (AFMC) Working Group has proposed a model for information exchange termed the Learner Education Handover (LEH) similar to learner handovers proposed by others however with the unique feature of input from the learner [15]. This model refers to the sharing of relevant information to facilitate a smoother transition for the learner proceeding from undergraduate medical education (UME) to postgraduate medical education (PGME). An ideal LEH focuses on the transfer of relevant information related to academic progression including medical knowledge, academic vulnerability, learner accommodation requirements, professionalism challenges, and health, emotional and social issues. In the context of the LEH the term accommodation [16] refers an adaptation or adjustment to the learning environment, curriculum format, or equipment that would assist the learner with any disability or health concerns (both physical and mental) to thrive as a medical learner.

An assumption for the LEH is that basic competencies/standards (entrust able professional activities [17–19]) have been met in order to proceed to residency and residency matching occurs prior to the information exchange. The handover is focused on providing a more comprehensive understanding of the learners, to enable them and their programs to achieve a smoother, transparent, robust transition, enhancing safety for the learner, residency program and ultimately for patients.

Examples of the difficulties encountered during the transition from medical school to residency include physical and mental health issues such the need for special equipment, extra exam time, duty hour reductions during clinical clerkship, modified call hours, personal coping readiness and other adequate psychosocial supports. These may be remediated by a learner handover by allowing information to be shared about the learner prior to starting residency so that appropriate alterations can be made by PGME and if necessary, residency programs. This is because it would allow for an early system of alerting programs while providing a medium for learners to disclose any concerns before starting residency. Given the controversies that exist around information sharing between UME and PGME programs, and that there are various stakeholders involved in a LEH process however, the purpose of this study was to determine the perceptions of key stakeholders regarding a national LEH process for medical learners in Canada after they have been matched to their residency program.

## Method

The AFMC LEH working group was comprised of student affairs leaders, medical students representing a national medical student organization, residents representing a national resident organization, postgraduate medical education deans, undergraduate medical education deans and a non-clinician medical education researcher. We decided to conduct a qualitative study as opposed to a quantitative study (survey) because we wanted to obtain insights from key stakeholders about the idea of a national LEH. We believed that conducting focus groups would help to uncover attitudes and allow for us to explore in depth the risks and benefits associated with a LEH. Most importantly, we wanted our participants to feel they had a “voice” in a national initiative.

Ethics board approval for this study was obtained from the University of Calgary Conjoint Health Research Ethics Board (CHREB) Ethics ID: REB15-1244. We developed the interview guide as a group. Given that the AFMC working LEH working group is comprised of key stakeholders themselves, the interview guide was developed over the course of several telephone meetings during which discussions were held about each question and edited accordingly. Working group members had the opportunity to comment on drafts of the interview guide between meetings via e-mail after liaising with members from the key stakeholder groups they represented. They would then bring their comments back to the working group. Of note, there was little overlap (n=2) between the members of the AFMC LEH working group and the actual participants of the focus groups.

The working group developed an interview guide on the proposed *content*, *process* and *format* of the LEH and stakeholder opinions were solicited about the use of the LEH. A caveat was that the LEH would be used for all Canadian medical graduates entering residency at all schools in Canada post residency program match.

A project administrator outside of the research team identified and contacted potential participants in stakeholder groups ensuring that all 17 medical schools from all provinces across Canada were included and a wide range of perspectives could be sought. Key stakeholders included medical students, senior and junior residents, program directors, student affairs leaders, UME Deans, PGME deans and Medical Regulatory Authority representatives from across Canada. Focus groups of 2-9 participants met, via teleconference, between April and August 2015 and were facilitated by author AK. In one case, an individual interview was held due to scheduling constraints. The interviewer used the interview guide developed by the working group for the focus groups and the one interview. They were audio recorded and transcribed verbatim. Focus groups were homogeneous with respect to key stakeholders to ensure there was no bias introduced by hierarchy, for example, residents and program directors. The facilitator ensured each participant had a chance to speak and voice their opinions about the LEH.

Recruitment was carried out with the assistance of administrative staff at the AFMC. Since the AFMC is comprised of all of the 17 faculties of medicine across Canada, e-mails were sent to the respective key stakeholder groups associated with each school. Additionally, recruitment of medical students and residents was carried out through the national organization representatives on the AFMC LEH working group. The recruitment of Medical Regulator Authorities which are based in each province was carried out in conjunction with a national organization that oversees the provincial bodies called the Federation of Medical Regulatory Authorities of Canada.

Focus group data were collected over the course of five months (April 2015 to August 2015) and concurrently transcribed. Data was first raw coded for broad themes by author AK and member checked the data with the working group key stakeholder representatives who took these themes to their respective groups. For example, the medical student representative on the working group who also represented a national medical school organization, took the themes back to the members of the national medical student organization.

Two authors (AK, MR) then independently analyzed the data. We approached each data analysis initially by line-by-line coding of transcripts and definitive concepts of the *content*, *format* and *process* of a LEH. A definitive concept refers precisely to what is common to text within a transcript, by the aid of a clear definition in terms of attributes [20]. Our interview guide informed such attributes.

We attempted to achieve theoretical saturation [21] within each homogenous group so as not to miss any new information from any one key stakeholder group. That is, the development of themes and the emerging theory in the analysis process during the initial raw coding took place as the criterion for additional data collection. This was driven by the notion of theoretical saturation. For instance, we held a focus group with postgraduate medical education deans but did not achieve theoretical saturation after the first focus group, so we held two other subsequent focus groups with postgraduate medical education deans. This was true for other key stakeholder groups as well.

We also attempted to achieve thematic saturation [21] during the data analysis phase with our definitive concepts of *content*, *format* and *process*. During this deductive approach, saturation was reached when the pre-determined codes by way of definitive concepts from the interview guide were represented in the data. This is similar to the idea of the themes being sufficiently supplied with examples in the data.

We maintained rigor and fostered reflexivity throughout the data analysis process through the use of memo writing as well as regularly scheduled telephone meetings between the coders. As non-clinical medical education researchers, we underwent an ongoing critique and critical reflection of our own biases and assumptions and how these have influenced all stages of the research process. We maintained dialogue and discussed divergent understandings when examining key stakeholder opinions of the LEH and provided a context in which we discussed our hidden beliefs and assumptions. For instance since both coders were based in different institutions in different provinces in Canada they brought their own unique experiences and biases when coding which were documented and discussed. We were also cognizant of their own bias towards supporting the LEH given our position in PGME Offices and connection with the AFMC (for author/coder AK and not author/coder MR). We also acknowledged our passion for the advocacy for learners and learner wellness as areas of research that we are involved in.

Data was coded the data using NVivo (QRS NUD*IST NVivo) qualitative software [22, 23]. We used a thematic analysis approach informed by principles of constructivist grounded theory [24] to analyze transcripts acquired during the interview, and focus groups. We used constructivist grounded theory, which is interpretivist [25] in nature. This means that the notion of a shared reality is interpreted by the researcher and that “...reality arises from the interactive process and its temporal, cultural, and structural contexts.” [26] Constructivist grounded theory rejects the notion of an objective reality. Instead it postulates that reality, society and the self are socially constructed by developing shared understandings through social interaction with others (also known as social constructivism) [27]. We believe that this approach facilitates a researcher’s understanding of how a shared reality is created and how meaning is developed through the social interactions with others within defined contexts, and for this study, engaging key stakeholders for their opinions on the LEH.

As the analysis progressed, the application of codes became more focused and connections between various concepts were identified. Axial codes were progressively developed throughout the analytical process, but were finalized during later stages of analysis. Throughout each phase of analysis, we implemented a quasi-constant comparative approach, in which both coding of transcripts and provisional framework development based on the *content*, *format* and *process* of a LEH occurred in a concurrent and iterative fashion [28]. During the final stage of analysis, we collapsed codes, which were found to be similar across both coders [29]. Discrepancies were discussed and a third author (LN) was asked to help reach a decision where discrepancies could not be resolved. We then deductively reapplied this framework to the data.

## Results

Sixty key stakeholders were recruited and included: medical students (*n*=8), junior and senior residents (*n*=13), Program Directors (*n*=8), Student Affairs Leaders (*n*=8), UME Deans (*n*=7), PGME Deans (*n*=9), and Medical Regulatory Authority Representatives (*n*=7). Table 1 shows the distribution of males and females across the key stakeholder categories. We do not report other demographic data such as location because this would allow participant identities to be compromised.

**Table 1:** Key Stakeholder Group and Gender Distribution

Key stakeholder group	Number of participants (n)	Gender
Medical Students	8	*n* = 5 males, *n* = 3 females
MRAs	7	*n* = 2 males, *n* = 5 females
PGME Deans	9	n = 7 males, *n* = 2 females
Senior and Junior Residents	13	*n* = 7 males, *n* = 6 females
Student Affairs Leaders	8	*n* = 4 males, *n* = 4 females
UME Deans	7	*n* = 5 males, *n* = 2 females
Program Directors	8	*n* = 3 males, *n* = 5 females

**Fig. 1:**
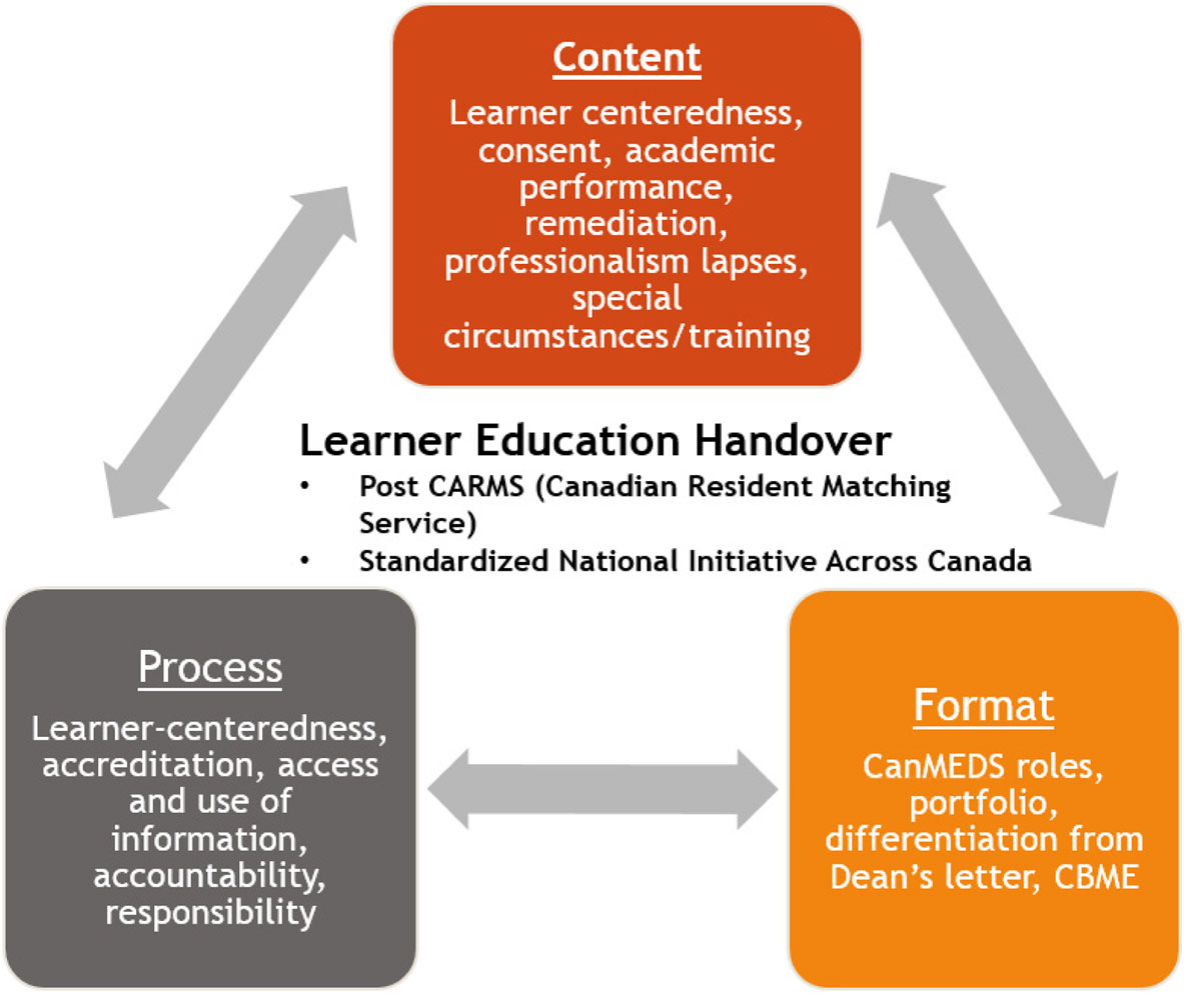
Themes of Content, Process and Format of the Learner Education Handover

Overall, stakeholders endorsed value in a national LEH document that would support learner transition from medical school to residency. Pertinent themes emerged in the areas of *content*, *process* and *format* and are described below. Stakeholders identified that the LEH must be learner centered which was defined as having learner involvement in reviewing the information provided (***content***), ensuring that the information would be used appropriately and as needed (***process***), and that it would be different from the existing MSPE/Dean’s Letter (***format***). Figure 1 shows the construct of an ideal LEH.

### Content of the LEH

Stakeholders reported that the LEH would be most useful if it included information on academic performance, professionalism lapses and special circumstances of training such as the need for an accommodation. There was no consensus whether the learner should have the ability to withhold consent over the release of information in the LEH. There was agreement that the process would apply to all learners (to normalize this as a routine process), and that the content be shared directly with the learner and reviewed by the learner when handover takes place. Most importantly medical students were in support of the idea of a LEH.

*“I’m definitely in favor of the idea. I think that there’s a lot of information that’s lost about a student between undergraduate and postgraduate medical education.” –* Medical Student 001–04

Medial students saw the utility of the LEH for learners that may require assistance in residency.

*“So I mean at the end of the day, I think we might all be able to think of specific students and there’s obviously concern about the may be ride that line of professionalism or ride that line of are you appropriate for medicine throughout medical school. And they slide through the matching process and they’ll hit the residency and then with that fresh sort of start the argument that they might be able also to slide through residency. And so, sort of potentially it’s bringing an awareness for postgrad teams to just have an awareness that this person needs certain deficits met or remediated so that they’re graduating as a competent staff physician at the end of it.”* – Medical Student 001–01

*“I think it would be good for the program director to know in advance students who are transitioning to residency who maybe in high likelihood of additional academic support in order for them to be successful at achieving their exams.” -* Medical Regulator Authority Representative - 002-03

Yet there was also acknowledgement that while the LEH would be for all incoming learners for those without concerns, there would be little information handed over.

*“And that it happens for everyone and maybe for some there’s very little handed over, but for the majority it may be that this student has exceptional skills in and no identified areas of challenge.”* Postgraduate Medical Education Dean 001–01

Key stakeholders also stated that the current information on prior performance is not of much value and specific information to help the learner would be beneficial.

*“We are interested in tailoring our educational programs to the strength and weakness of the resident. In other words, giving them the opportunities to make sure to enhance their strengths. And giving them opportunities to remediate their weaknesses. And essentially, the way they come right now, they all come in equally. We get a transcript that is of very little value. We don’t get any specifics and we would change the education that would ultimately be better for the learner if we knew a little better where they had more success or more difficulty in the earlier part of their training.”* Program Director 003–01

There was also emphasis that the content of a LEH would have to be transparent to the learner.

*“I think as long as the students are very aware of what is being revealed and handed over, then they know what’s coming. But there’s no way we can do the medical details of things. If there’s an accommodation that’s going to affect the way they train, I think they have to be aware that that would be handed over.”* Program Director 001–02

Overall, key stakeholders believed the LEH would be of value as an early alert system.

*“ … there may be patient safety related concerns that were brought up during that student’s time in medical school, which were not included in their interview application package for whatever reason. And that are red flags for going forward.” –* Resident 002–02

*“Well, I think in some of the bigger programs with many residents coming into the same program, if you do have certain issues or things that may be able to be accommodated early or worked on early, they’re not caught because you just kind of fly under the radar and then you may have to write exams and all of this other stuff if someone is not ready. And that could have been mitigated earlier, so I think without kind of knowing anything about the people coming into your program, you may miss the opportunity to help them early.” –* Resident 003–10

*“Of course from the postgraduate program’s point of view it’s almost a perfect starting point to have a defined baseline for your trainee …”–* Student Affairs Leader 001–04

### Process for the implementation of the LEH

Stakeholders indicated that the process of the LEH should be learner-centered. Transfer of information from UME to PGME should involve the learner. There was general consensus that PGME would act as the gatekeeper for the information ensuring that key individuals in the respective programs would be informed only if there were learner deficits that could impact the learner and residency program. Further consideration as to how the information would be used appropriately also emerged. There was no consensus as to whether the LEH might become part of a future accreditation process.

There was discussion that the implementation process would require faculty development with respect to how to use the information.

*“Without a very well-rounded faculty development initiative for program directors in particular, but also for training committees and faculty, you know there is a risk that the information could be used in a way that’s detrimental to the student and not in the spirit of the intended educational programming.”* Postgraduate Medical Education Dean 001–03

Regarding the information sharing process, medical students were often hesitant about how the information would be shared.

*“But I do feel like entering residency is more like an apprenticeship, so it’s more like a job. And in that sense I feel nervous about any documents being sent to the residency program that weren’t a part of the application process.”* Medical Student 002–01

There were also concerns as to how the information will be used once shared.

*“I think it may help the student themselves at the postgrad level if we can know what they need to be helped about. However, I think we should need to know if it’s going to be implemented at some point, how the information will be used. Will it be kept for the program director? We’re a bit afraid of forward feeding for the student if some information is shared with everyone in the program before they arrive. So that’s something that needs to be surrounded by clear consent about what will be done about the information, so we know better what we should and could transfer.”* Undergraduate Medical Education Dean 001–06

The process of information storage was also elaborated upon. Essentially PGME could hold the information and only relay it to program directors when necessary.

*“The PGME Office needs to hold the information and then use it on a case-by-case basis with interactions with probably the program director and whoever else needs to be involved, depending on the nature of the information.” -* Postgraduate Medical Education Dean 003–08

*“I was thinking about the process of after the information was received and PGME holds the information, rather than it being distributed directly to the program directors. Because I think there sometimes can be what should be disclosed to whom questioned when the information comes in.” -* Postgraduate Medical Education Dean 001–05

*“I think it would be good for the program director to know in advance students who are transitioning to residency who maybe in high likelihood of additional academic support in order for them to be successful at achieving their exams.” -* Medical Regulator Authority Representative - 002-03

The importance of standardizing the process was also highlighted. This would need to occur before accreditation.

*“There is going to have a period where you implement it at first to make sure that it works and can be standardized and then you can start talking about accreditation I think.”* Resident – 003-04

*“I think the information in some kind of consistent standardized format goes from the undergraduate office to the postgraduate office where the resident has been accepted in the postgraduate training program. And at that level, we, that college and that university then have a discussion on a need to know basis.” -* Medical Regulator Authority Representative - 002-04

Some stakeholders believed that the LEH as an accreditation requirement would not be necessary or would require further buy-in before being considered as an accreditation standard.

*“….accreditation is about the process within the medical school and the assessment and teaching processes and the student oversight in the medical school. It does not speak to where graduate information goes, nor should it.”* - Undergraduate Medical Education Dean 002–02

*“But my initial thought is that that would be quite difficult at the moment until we got schools to sort of buy-into the process. And I don’t necessarily think that right now the way it’s being presented it would be something that’s necessary for a school to need accreditation standards. I’ve been involved with the accreditation practice quite significantly throughout my four years at medical school and I know that there’s a lot of standards that need to be met and schools have a lot of responsibilities. So I think right now in this initial phase, I don’t think that that’s necessary and I don’t necessarily think that putting a lot of time and effort into that sort of transition phase to make it up to accreditation standards is necessary at the moment.”-* Medical Student 001–03

### Format of the LEH

Stakeholders agreed that the LEH should be different from a MSPR/Dean’s letter and that the UME Dean would provide it. The LEH format of presentation was discussed including the idea of a learner-directed portfolio. It was agreed that alignment with the evolving approach to competency-based medical education and reference to the CanMEDS roles [18, 30] is important although the CanMEDS roles may not cover all aspects of the LEH. CanMEDS is an educational framework that describes the abilities physicians require to effectively meet the health care needs of the people they serve [18, 30].

Stakeholders believed the format of the LEH would benefit as a summative piece of information that goes beyond the MSPR.

*“I don’t think as postgrad deans, we necessarily need every blow-by-blow in the analysis, which is formative. But I do think there has to be some kind of a summative description or summary of the undergraduate experience. And so, currently that summative piece is the MSPR, which is not desirable from both undergrad needs and postgraduate needs. I think some newer rendition of a summative piece that will replace the MSPR perhaps is what we’re looking for.” –* Postgraduate Medical Education Dean 003–02

There were concerns however with respect to the format the LEH would have with respect the information shared from UME.

*“I’m having trouble seeing how, as you say, in undergrad we could generate that in an accurate or reliable way.”* - Student Affairs Leader - 001-005

*“I think that certainly makes a lot of sense if everyone is speaking the same language. I can’t off the top of my head think of a better way to organize the information. Now that I think about it there needs to be some sort of like miscellaneous category. I think that maybe CanMEDs is a little prescribed and doesn’t adequately account for the breadth of what may be in a handover document.” –* Medical Student −003-07

*“Where I’m a little hesitant is how you would quantify whether or not somebody met all those CanMEDs roles during the handover process from medical school. How would you say somebody was a communicator?”-* Resident −003-13

*“I would agree. It’s not all going to fit in a CanMEDS sort of framework. I mean certainly even things like personal health issues that need to be addressed for the learner.” –* Postgraduate Medical Education Dean −003-13

### Risks and benefits of the LEH

**Fig. 2:**
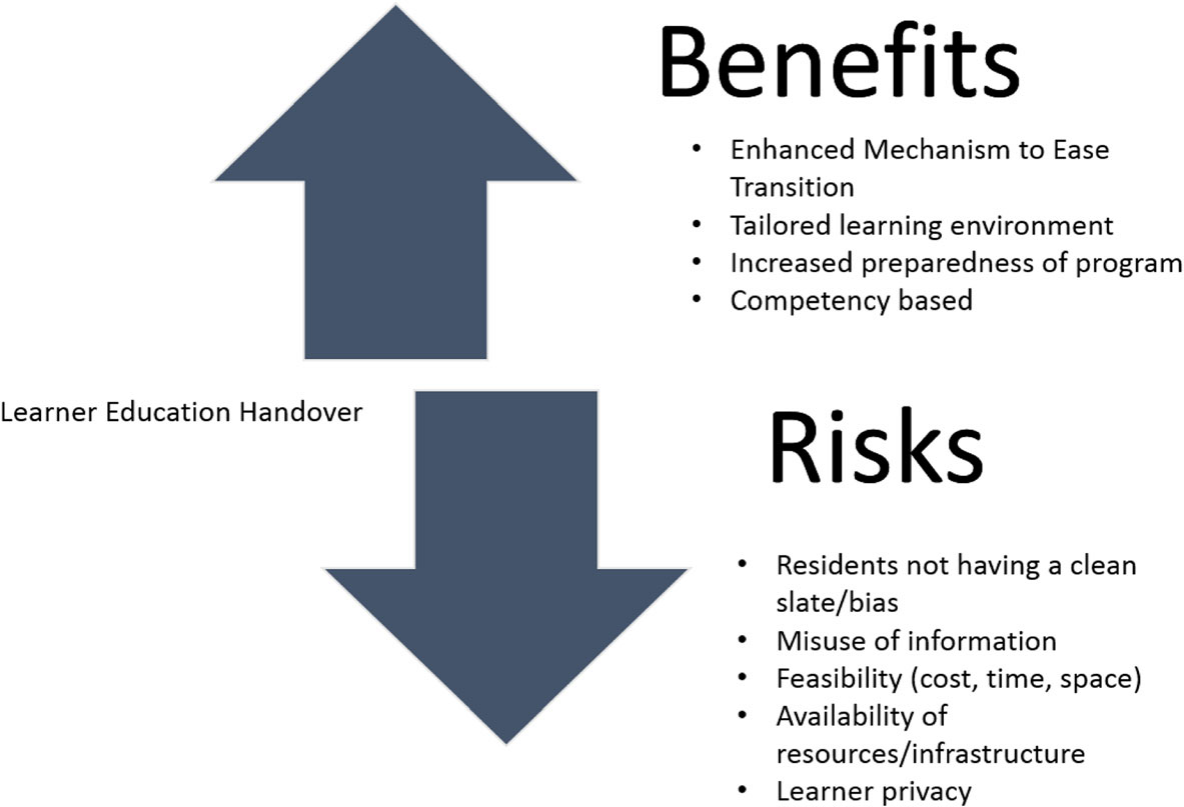
Themes of Risks and Benefits of Learner Education Handover

Potential risks and benefits to having an LEH emerged (Fig. 2).

An identified perceived risk to the LEH was the possibility that learners may be subjected to bias thereby being unable to start their residency free from previously resolved issues which key stakeholders referred to as a “clean slate” or a “fresh start”. Other risks of the LEH identified were *i)* the potential lack of security resulting in the misuse of information, *ii)* the feasibility of the implementation of LEH and *iii)* whether there is adequate infrastructure in place for a standardized national initiative.

Stakeholders also reported several perceived benefits to having a LEH, resulting in an enhanced transition from UME to PGME. For example, information shared could improve patient safety through enhanced quality assurance, promote the development of expected professional behaviors, continuity of mentorship, resident wellness, and .prevention of relapses. The LEH could facilitate self-directed learning, supported by the individualized learning plan.

With respect to the residency program, a perceived benefit includes the use of a learner-centered transition plan. Respondents indicated that the LEH would also allow the program to be more adequately prepared for the learner. Quotes reflecting the risks and benefits can be found in Table 2.

**Table 2:** Key Stakeholder Opinions about the Risks and Benefits of a Learner Education Handover (LEH)

Risks of the LEH	Benefits of the LEH
*“There are those occasional circumstances where you know, the sharing of information might make that fresh start difficult when a fresh start is actually the most useful thing. I think most of the time that’s actually not the case, but if we’re thinking about risks that’s what occurs to me.” – Postgraduate Medical Education Dean – 003-09*	*“The biggest benefit of a handover is an individualized teaching plan. I mean it can also be used in negative ways.” - Resident – 003-10*
*“I think that residency should be a clean slate for medical students and one of the reasons that I feel that way is because in certain situation or certain circumstances medical students may thrive or may not do so well. And the whole idea of residency is the change of environment.”- Medical Student – 001-01*	*“I mean what I like is I would like the idea of there being some continuity. But I don’t know how we can practically do it with a handover from every medical student to every training program. But I do like that continuing mentorship, continuing fostering certain skills. You know if there’s professionalism and mentoring that’s happening that that gets continued.” – Student Affairs Leader – 001-07*
*“In the UME program difficult students who come to our attention and sometimes they start to evoke negative emotions in us and similarly, there’s some students we feel extremely positive about. At the end then there’s a whole swath of people in the middle, so there’s always bias in these assessments.” - Undergraduate Medical Education Dean – 002-05*	*“I think one of the things that handover should be helpful for is allowing programs to know what level of responsibility they can be assigning to people within those early weeks and months of their residency training. What skills do they have? And what things are they not really in a position to be able to do independently? And I think ultimately that should be in the interest of better patient care.” Postgraduate Medical Education Dean – 002-06*
*“I think for the learner’s standpoint I think that their biggest concern would be risk of bias. And they wouldn’t want for people to have presupposed or pre-judge them based on something which they believe or have in fact resolved in their previous training. So how that information is then disseminated or circulated once it’s in the hands of a program director I think is a key point. Just as we deal with forward feeding/orienting preceptors to learners within residency, I think it’s the same at that transition point. So it is a very tricky line between sort of the negative connotations of forward feeding as opposed to the more positive connotation of having a positive or responsive or appropriate learning environment for a learner. How exactly that balance is met I think is exactly why we’re having this conversation.” – Program Director – 002-02*	*“Well, if its objective information that has been validated and is being transmitted with the intention of allowing the student to function better in the new environment. That’s going to be clear to everybody. I mean it’s going to be documented and the particular accommodations are going to be already understood. And the student is going to be aware that that information is going forward and why.” Undergraduate Medical Education Dean – 001-03*
*“When you’re handing over that kind of information you have to recognize that the residency programs don’t really know the resident yet, so it’s hard to really put that information in context of their overall person and their skills and their performance.” Resident – 001-02*	*“…we know right away we’re going to have applicants who are misrepresenting their file. We have applicants that are going to be practicing without a license and getting in trouble right off the bat. We have individuals who aren’t going to properly disclose emotional and anxiety issues who are going to end up on physical leaves* ^a^ *right away.” - Medical Regulatory Authority Representative – 003-01*

^a^Leave of absence due to illness

## Discussion

This study is the first to engage medical education stakeholders in a discussion regarding the use of an LEH to share medical learner information between UME and PGME to ease the transition from medical school to residency. While the idea of a LEH was viewed positively, potential risks were identified related to bias, security and feasibility.

Our study provides preliminary insights into a national LEH process and whether it would be of value to learners to ease the transition from UME to PGME. Stakeholders reported that the LEH would be useful for both the learner and the program. Caveats with respect to the process would allow a learner-centered approach while focusing on patient safety, resident wellness and professional behavior. Both the learner and UME would complete documentation for the LEH. An identified risk is of potential bias towards the learner and the loss of the possibility of a “clean slate” when beginning residency. The most frequently recognized benefit to the process was enhanced program preparation for particular learner needs.

While the concept of a formalized LEH has support, the nature of its *content*, *process* and *format* warrants further investigation. With respect to *content*, key stakeholders agreed that an LEH would be most useful if both the learner and UME contribute content, which could include strengths of the learner to the residency program. The *process* for the LEH requires accountability with respect to the transfer and use of information as well as the acknowledgement that this process is ultimately to help the learner.

Timing of the handover must be after the residency match process so as to avoid any hesitation by learners in sharing their information for fear it may impact their residency program match. While it might be beneficial to have a LEH before the matching process, this would pose logistical problems since the Canadian matching process is independent of PGME programs in Universities. Furthermore, to ensure learner buy-in, a post-match LEH can be seen as more learner-centered. Regarding *format*, the LEH should also provide information that goes above and beyond the MSPR/Dean’s letter that would help assist PGME and programs to tailor learning plans for incoming residents if there is a need. Furthermore, PGME would be the gate keeper for the LEH information and would only share it with programs on a “need to know” basis. Incorporating CanMEDS roles while helpful would not be sufficient for a national LEH. Other areas to be included are the wellness of the learner and the learner’s preparedness for residency. The conceptual framework of the LEH warrants further key stakeholder input and investigation along with future beta and pilot testing of an LEH to ensure it provides functional information for residency programs while meeting learner needs.

Unlike the studies by Warm et al. [5] and Sozener et al. [6], we consulted key stakeholders regarding the *content*, *process* and *format* of a LEH. The results of this study demonstrate value in developing a handover tool specifically for information sharing purposes in addition to existing assessments. Another strength of our study included representation from stakeholders across the country, increasing the generalizability of our results and rigorous study methods to ensure that the LEH is based on key stakeholder input promoting support and buy-in for such an initiative. Our study also highlights the need for learner-centeredness for potential national level policy initiatives. As such a learner-centered LEH should consider a holistic learner approach by creating a conducive learning environment that is free of harassment, promotes a safe environment for students to ask questions, or voice opinions, supports the psychological and social characteristics of learners, embraces learner diversity and individual learning needs while recognizing learner strengths and activities in areas beyond those traditionally valued in future physicians [14]. These may include life experience and leadership attributes, which are not afforded the same level of influence as clinical knowledge when entering residency [31].

There are several limitations of this study. First, participant stakeholders who consented to participate in this study may be more positively inclined because they perceive their vested interest in the LEH and our results may not accurately represent key stakeholder groups more broadly. Second, while we had members from the key stakeholder representatives (for example, medical students and residents) on the LEH working group as proxies and vehicles for member checking our work with the LEH, this does not afford the same opportunities as member checking with the study actual participants. Third, the AFMC deemed the LEH as an important initiative for medical schools across Canada, and as such the working group and all stages of this study may have a biased lens in favour of an LEH given that the working group already sees value in the LEH. We tried to mitigate this by having an external coder during data analysis and by fostering reflexivity throughout the however other strategies could have also been used such as member checking with actual participants or conducting a mixed methods study. For example, we could have developed a survey on key stakeholder opinions of the LEH to obtain a broader range of responses followed by interviews conducted and analyzed by independent researchers who were not part of the AFMC. This may have also allowed for the triangulation of data from various sources. Last, we did not sample clinical educators/preceptors as part of our key stakeholder consultations. This would be useful given that information in the LEH could be shared with residency programs and as such the clinical educator/preceptors involved in the teaching and assessment of residents. We chose not to include clinical educator/preceptors for this study because we were exploring the concept for LEH and saw clinical educator/preceptors as important key stakeholders but further downstream in the implementation process of the LEH.

Concerns about bias with respect to previous performance information in a LEH were plentiful in our study data and seen as a major threat to the LEH being learner-centered by key stakeholders. Future studies should consider program director and clinical educator/preceptor perceptions of the LEH and previous performance information bias. Additionally further research needs to ensure that learner handovers function appropriately in medical education and ultimately help ease learner transition from medical school to residency.

## Conclusions

Learner handovers are garnering much attention in the era of competency based medical education. However much of the learner education handover models that exist today focus on clinical performance without input from the learner. A new stakeholder-informed information sharing process termed called the Learner Education Handover (LEH) should be tested to ensure feasibility, utility and benefits. The LEH must be learner-centered, respecting learner privacy, while facilitating a smooth transition from undergraduate to post-graduate medical training, promoting learner wellness, professionalism, and performance.

## Abbreviations

AFMC: Association of Faculties of Medicine of Canada
CanMEDS: Canadian medical education directions for specialists (framework)
CHREB: Conjoint Health Research Ethics Board
LEH: Learner Education Handover
MSPE: Medical Student Performance Evaluation
MSPR: Medical Student Performance Record
PGME: Postgraduate Medical Education
UME: Undergraduate Medical Education
